# Contribution of factor H-Binding protein sequence to the cross-reactivity of meningococcal native outer membrane vesicle vaccines with over-expressed fHbp variant group 1

**DOI:** 10.1371/journal.pone.0181508

**Published:** 2017-07-25

**Authors:** Arianna Marini, Omar Rossi, Maria Grazia Aruta, Francesca Micoli, Simona Rondini, Serafina Guadagnuolo, Isabel Delany, Ian R. Henderson, Adam F. Cunningham, Allan Saul, Calman A. MacLennan, Oliver Koeberling

**Affiliations:** 1 Institute of Immunology and Immunotherapy, College of Medicine and Dental Sciences, University of Birmingham, Birmingham, United Kingdom; 2 GSK Vaccines Institute for Global Health (GVGH), Siena, Italy; 3 GSK Vaccines, Siena, Italy; 4 Institute of Microbiology and Infection, College of Medical and Dental Sciences, University of Birmingham, Birmingham, United Kingdom; Public Health England, UNITED KINGDOM

## Abstract

Factor H-binding protein (fHbp) is an important meningococcal vaccine antigen. Native outer membrane vesicles with over-expressed fHbp (NOMV OE fHbp) have been shown to induce antibodies with broader functional activity than recombinant fHbp (rfHbp). Improved understanding of this broad coverage would facilitate rational vaccine design. We performed a pair-wise analysis of 48 surface-exposed amino acids involved in interacting with factor H, among 383 fHbp variant group 1 sequences. We generated isogenic NOMV-producing meningococcal strains from an African serogroup W isolate, each over-expressing one of four fHbp variant group 1 sequences (ID 1, 5, 9, or 74), including those most common among invasive African meningococcal isolates. Mice were immunised with each NOMV, and sera tested for IgG levels against each of the rfHbp ID and for ability to kill a panel of heterologous meningococcal isolates. At the fH-binding site, ID pairs differed by a maximum of 13 (27%) amino acids. ID 9 shared an amino acid sequence common to 83 ID types. The selected ID types differed by up to 6 amino acids, in the fH-binding site. All NOMV and rfHbp induced high IgG levels against each rfHbp. Serum killing from mice immunised with rfHbp was generally less efficient and more restricted compared to NOMV, which induced antibodies that killed most meningococci tested, with decreased stringency for ID type differences. Breadth of killing was mostly due to anti-fHbp antibodies, with some restriction according to ID type sequence differences. Nevertheless, under our experimental conditions, no relationship between antibody cross-reactivity and variation fH-binding site sequence was identified. NOMV over-expressing different fHbp IDs belonging to variant group 1 induce antibodies with fine specificities against fHbp, and ability to kill broadly meningococci expressing heterologous fHbp IDs. The work reinforces that meningococcal NOMV with OE fHbp is a promising vaccine strategy, and provides a basis for rational selection of antigen sequence types for over-expression on NOMV.

## Introduction

*Neisseria meningitidis* is a leading cause of bacterial meningitis worldwide, and recurrent epidemics in Sub-Saharan Africa [1, 2]. An important meningococcal virulence factor and vaccine antigen is factor H-binding protein (fHbp). fHbp is an outer-membrane surface-exposed lipoprotein, expressed by almost all meningococcal strains, albeit at varying levels [3–7]. It binds human factor H (fH), a negative regulator of the alternative pathway of the complement cascade, allowing meningococci to escape innate immunity [8, 9]. Antibodies directed against fHbp are bactericidal, and can both activate the complement cascade, and block the recruitment of fH by bacteria [5, 6, 9–13]. fHbp is included in licensed protein-based vaccines against group B meningococcus [14, 15].

On the basis of amino acid sequence, fHbp is classified into two sub-families (A and B) [10], or three variant groups (v.1, v.2, v.3) [5]. fHbp can be further divided into more than 1,000 sub-variants, with each individual amino acid sequence distinguished by an identification number (ID); the majority of these sub-variants are included in variant group 1, which currently contains 580 IDs, while variant groups 2 and 3 have 215 IDs each. Sera raised against recombinant fHbp generally are bactericidal against strains expressing fHbp belonging to the same variant group, with limited cross-reactivity between variant groups. However, fHbp sequence diversity within each variant group limits breadth of bactericidal activity [3, 5, 6, 10, 16, 17].

Native Outer Membrane Vesicles (NOMV) are membrane blebs that can be shed naturally by Gram-negative bacteria [18]. Meningococcal detergent-extracted OMV (DOMV) have been used safely and effectively as vaccines in humans for almost 30 years but provide strain-specific protection [19, 20]. Previous studies have shown that meningococcal NOMV with over-expressed (OE) fHbp v.1, induce antibodies in mice with broader bactericidal activity against African isolates, than antibodies induced by DOMV or recombinant fHbp [21, 22]. However, the specific contribution of the fHbp sequence ID to the cross-reactivity of antibodies induced by NOMV OE fHbp has not been extensively studied.

We did not focus our attention on the entire fHbp protein sequence, as this would have considered variations at positions not necessarily immunological relevant. However, the fH-binding region is surface-exposed, and variation is likely to be subjected to a balance between opposing selection pressures from the human immune system and functional constraints. Therefore, we performed a comparative analysis of the region of fHbp involved in fH binding, likely to be an important target for protective antibodies. We studied the specificity and cross-reactivity of anti-fHbp antibody responses elicited by NOMV over-expressing different fHbp ID types, by investigating the relationship between the amino acid sequence of this region of fHbp and the extent of cross-reaction of antibodies induced by NOMV with over-expressed fHbp. Because a vaccine that can protect against multiple serogroups of *N*. *meningitidis* is particularly needed for Sub-Saharan Africa, we compared cross-reactivity of sera elicited by NOMV OE different fHbp ID types belonging to variant group 1 (the most prevalent fHbp variant group among Africa strains [16]), against serogroups of meningococcus that are prevalent in Africa.

## Materials and methods

### fH-binding site analysis on fHbp

**Fig 1:**
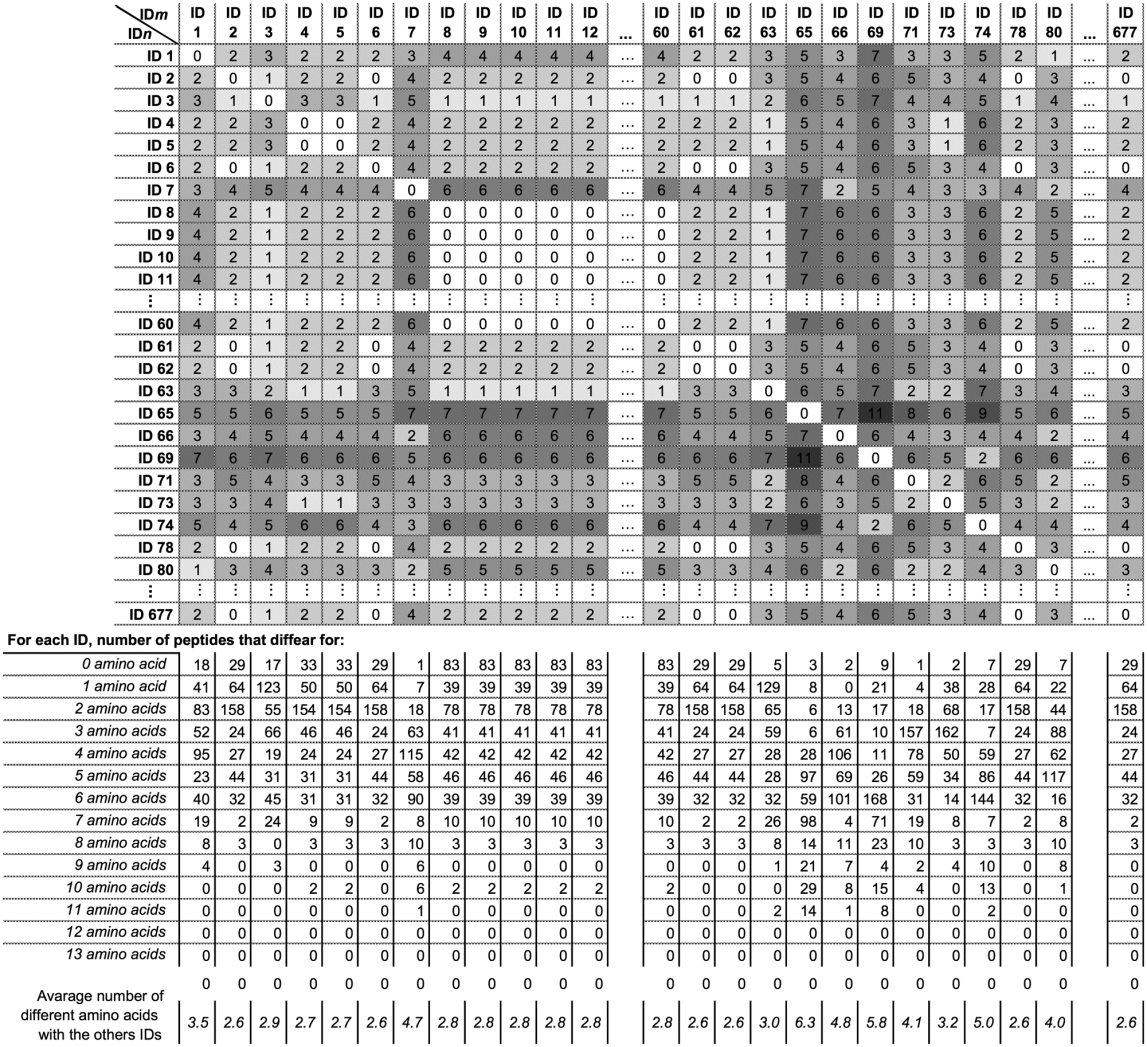
Section of matrix for pairwise comparison of fH-contact residues on fHbp v.1 IDs. Each cell_*mn*_ contains the number of different amino acids in the fH-binding site between ID_*m*_ and ID_*n*_. The darkness of shading of each intersection corresponds to the number of differences, with white = 0 differences. In the table below the matrix, for each ID_*m*_ the number of fHbp v.1 peptides differing for an increasing number of amino acids is given, together with the average number of different residues between ID_*m*_ and all other v.1 peptides. IDs belonging to v.1 between 12 and 60, and between 80 and 677 are not shown (full matrix available in S2 Table).

We mapped the residues of fHbp that are in contact with fH on the published crystal structure of the fHbp v.1-fH complex [23, 24], using the publicly available software Cn3D. Parameters were set to identify residues on fHbp that are at a maximum distance of 5 Å from the fH molecule, revealing 48 amino acids as points of interaction between fHbp and fH. We aligned the 383 unique fHbp v.1 peptide sequences published on the Neisseria.org database [25], and identified the 48 amino acids contributing to the fH-binding site on each sequence, using fHbp ID 1 as the reference sequence (S1 Table). To compare the number of different amino acids between each pair of the 383 IDs, we created a *mn* matrix in which each cell contains the number of amino acid differences between sequence *m* and sequence *n* (Fig 1 and S2 Table).

### *Neisseria meningitidis* strains and generation of mutants

The parental wild type strain, used for generating *N*. *meningitidis* mutants for NOMV production, has been described by us previously [21]: it is a serogroup W, ST11 clone, isolated in 2004 in Ghana, expressing fHbp variant group 2 ID 23, and PorA serosubtype P1.5,2 [21]. To generate mutants over-expressing *fHbp v*.*1*, the native *fHbp v*.*2* gene was deleted and replaced by an erythromycin resistance cassette [5]. The resulting *fHbp v*.*2* KO mutant was subsequently used to generate a panel of isogenic strains, over-expressing four different *fHbp v*.*1* ID types. *fHbp v*.*1* genes were amplified by PCR from genomic DNA of different meningococcal strains (ID 1 from MenB MC58; ID 5 from a MenA isolate; ID 9 from a MenW isolate, and ID 74 from a MenX isolate), with primers as shown in Table 1. The R41S mutation, known to reduce the human fH binding to fHbp [26], was introduced in the full-length *fHbp* genes using the Q5 Site-Directed Mutagenesis Kit (NEB), following the manufacturer’s protocol. *fHbp* genes were inserted into the group W capsule biosynthesis locus, in the place of *synXBCD*, under the control of a strong synthetic promoter [27]. A MenW control strain devoid of both fHbp expression and capsule biosynthesis was produced by deleting capsule biosynthesis genes in the MenW *fHbp* KO mutant, as described before [21].

**Table 1:** Sequences of primers used in this study.

Purpose	Primer designation	5′-3′ primer sequence
Amplification of up-stream recombination site of capsule biosynthesis locus	Cps_upF	TCCCCCCGGGTATCGCCAACAAACGGCACAG
	Cps_upR	GCTCTAGATTTCGATTAAGTGCTATAATTAGGCC
Amplification of down-stream recombination site of capsule biosynthesis locus	Cps_doF	GCCAATGCATCAATATGCTGCCATTACTCC
	Cps_doR	GGACTAGTGTTTGTTTGCCGCATGCTAATGCC
Amplification of *fHbp* ID 1, 5, 74	fHbp_fw	CGCGGATCCCATATGAATCGAACTGCCTTCTGCTGCC
	fHbp_rev	CCATTGTGAAAATGCCGTC
Amplification of *fHbp* ID 9	ID9_fNde	CGCGGATCCCATATGAACCGAACTACCTTTTTCTGCC
	fHbp_rev	CCATTGTGAAAATGCCGTC

### NOMV preparation

NOMV were prepared as previously described [21]: bacteria were grown at 37°C, 5% CO_2_, in 50 mL of medium (containing yeast extract, casaminoacids, and lactic acid), to stationary phase. Cells were harvested by centrifugation at 4,000 *g*, and the supernatant was filtered through a 0.22 μm pore size Stericup (Millipore, Billerica, MA, USA). NOMV were collected by ultracentrifugation of the filter-sterilized culture supernatant (186,000 *g*, 2 h, 4°C, using Beckman polystyrene tubes). After ultracentrifugation, the NOMV-containing pellet was resuspended in phosphate buffered saline (PBS), and sterile-filtered through a syringe filter with 0.22 μm pore size. NOMV concentrations were determined based on the total protein content, as measured by Lowry assay, in comparison to a standard curve obtained with bovine serum albumin (BSA) (Sigma-Aldrich, St. Louis, MO, USA) [28]. For protein analysis, NOMV were separated by SDS–PAGE using NuPAGE 12% Bis-Tris Protein Gels, and NuPAGE MOPS SDS Running Buffer (Invitrogen, Carlsbad, CA, USA). Total proteins were stained with Coomassie Blue stain (Sigma-Aldrich). fHbp was detected by Western blot using a polyclonal antibody raised in mice against recombinant fHbp v.1 ID 1. All the NOMV used in this study are listed in detail in Table 2.

**Table 2:** List of NOMV used in this study.

NOMV designation	Parental strain	Mutations in the NOMV-producing strain	Description
NOMV MenA wt*	MenA	none	Native expression of fHbp v.1 ID 5
NOMV MenW wt*	MenW	none	Native expression of fHbp v.2 ID 23
NOMV fHbp KO	MenW	*cps* KO; *fHbp v*.*2* KO	fHbp KO control
NOMV OE ID 1	MenW	*cps* KO; *fHbp v*.*2* KO; OE *fHbp* ID 1_R41S_	Over-expression of fHbp ID 1_R41S_
NOMV OE ID 5	MenW	*cps* KO; *fHbp v*.*2* KO; OE *fHbp* ID 5_R41S_	Over-expression of fHbp ID 5_R41S_
NOMV OE ID 9	MenW	*cps* KO; *fHbp v*.*2* KO; OE *fHbp* ID 9_R41S_	Over-expression of fHbp ID 9_R41S_
NOMV OE ID 74	MenW	*cps* KO; *fHbp v*.*2* KO; OE *fHbp* ID 74_R41S_	Over-expression of fHbp ID 74_R41S_

*NOMV were used as controls in analytical testing

### Cloning, expression, and purification of recombinant fHbp

The four gene sequences encoding the fHbp ID types over-expressed in the meningococcal mutants were cloned without the signal peptide sequence, and with a C-terminal hexahistidine-tag, into the expression vector pET21b (Novagen, Madison, WI, US). All four sequences contained the R41S mutation. Recombinant proteins were expressed and purified from *E*. *coli* BL21(DE3) (Novagen, Madison, WI, US), as described elsewhere [5].

### Biophysical analyses of purified NOMV

NOMV preparations were analysed by HPLC-SEC with a TosoHaas TSK gel 6,000 + 4,000 PW columns equilibrated in PBS, and with in-line UV, fluorescence emission, and MALS (Dawn Heleos II, Wyatt) detectors. 80 μL of samples, containing 0.1 mg/mL total protein, were injected and eluted with a flow rate of 0.5 mL/min in PBS. UV, fluorescence emission, and multiangle light scattering (MALS) data were collected and analysed using Empower 3 and ASTRA 6 software (Wyatt Technology). Light scattering analysis was done using “Zimm” model with fit degree 1.

Dynamic light scattering (dls) measurements were performed with a Malvern Zetasizer Nano ZS (Malvern, Germany), equipped with a 633-nm He-Ne laser and operating at an angle of 173°. The software used for collecting and analysing data was the Setasizer 7.11 from Malvern. 40 μL of each sample were measured in ZEN 0040-disposable micro cuvette (Malvern). The measurements were done with an automatic attenuator, and at controlled temperature of 25°C. For each sample, 10–15 runs of 10 s were performed, with 3 repetitions. The intensity size distribution, the Z-average diameter, and the polydispersity index (PdI) were obtained from the autocorrelation function using the “general purpose mode”. Default lower threshold of 0.05 and upper threshold of 0.01 were used. Viscosity and refractive index of PBS at 25°C were used for data analysis.

### Mouse immunisation

4–6 weeks old female CD-1 mice were obtained from Charles River Laboratories (Wilmington, MA, USA). Eight mice per group were immunised twice, 4 weeks apart, by intraperitoneal injection. Serum samples were obtained from bleeds at 2 and 4 weeks after the first dose, and 2 weeks after the second dose. On the basis of previous studies [16, 21], doses were chosen as follows: mice were immunised with 1 μg of NOMV OE fHbp (containing 85 ng of fHbp protein), or 5 μg of NOMV fHbp KO, or 20 μg of recombinant protein. 20 μg of recombinant fHbp is the dosage selected for a maximum response; 1 μg of NOMV OE fHbp is sufficient to elicit a potent antibody response [21], but for NOMV fHbp KO we used the higher dose of 5 μg to enhance the immune response from the negative control. All vaccines were adsorbed on 3 mg/mL Alhydrogel (Brenntag), in a formulation containing 10 mM Histidine and 0.9 mg/mL NaCl. Sera were stored at -80°C until use.

### Ethical statement

The animal protocol was approved by the Animal Welfare Body of Novartis Vaccines, Siena, Italy, and by the Italian Ministry of Health (Approval number AEC201309). The mouse immunisation experiments were performed at the Toscana Life Sciences (TLS), Siena, Italy, in compliance with the relevant Italian guidelines (Italian Legislative Decree n.116/1992). Mice were housed in appropriate plastic cages, and provided with food, water, and wooden litter. Each cage was maintained in ventilated cabinets with constant air flow and 12 hours of light per day. Animals were monitored at least twice a day during the study; during the two days following each immunisation, particular attention was dedicated to signs of good health and abnormalities at the site of injection. Mice were euthanized with CO_2_ after anesthetization.

### Serological analysis

Anti-fHbp ID 1, ID 5, ID 9, ID 74 serum IgG antibodies were measured by enzyme-linked immunosorbent assay (ELISA) as described elsewhere [29]. Each recombinant fHbp ID was used for ELISA plate coating at a concentration of 1 μg/mL. Mouse sera were diluted 1:100, 1:4,000, or 1:160,000 in PBS containing 0.05% Tween 20 and 0.1% BSA. ELISA units were expressed relative to a mouse anti-fHbp ID 9 IgG standard curve. One ELISA unit was defined as the reciprocal of the standard serum dilution that gives an absorbance value of 1 in the assay; each mouse serum was tested in triplicate on different plates. Data are shown as scatter plots of the mean of individual mouse ELISA units, and the geometric mean is shown for each group.

Serum bactericidal antibody response against meningococcal clinical isolates was measured on individual sera as described before [21]. 20% final concentration of Baby Rabbit Complement (CEDARLANE, Canada, lot number 6332), screened for lack of bactericidal activity against the target strains, was used as complement source in the assay; this was chosen instead of human complement because of the large amount of complement required to test individual mouse sera. Bactericidal titres were defined as the reciprocal extrapolated serum dilution resulting in 50% killing of bacteria, after 60 min incubation at 37°C, compared to the mean number of bacteria in five control reactions at time 0. Origin and characterisation of the strains tested are summarized in Table 3.

**Table 3:** Characteristics of *N*. *meningitidis* strains used in in this study.

Designation	Serogroup	Year of isolation	Country	CC [ST]^a^	PorA subtype	fHbp variant group and ID^b^	fHbp expression (%)^c^
Parent NOMV vaccine strain	W	2004	Ghana	11 [11]	P1.5, 2	v.2, 23	ND^d^
Test strains for SBA							
MenA 1	A	2009	Nigeria	5 [7]	P1.20,9	v.1 ID 5	107
MenA 2	A	2006	Burkina Faso	5 [2859]	P1.20,9	v.1 ID 5	52
MenA 3	A	2007	Burkina Faso	5 [6035]	P1.20,9	v.1 ID 5	65
MenW	W	2009	Cameroon	175 [2881]	P1.5–1,2–36	v.1 ID 9	1
MenX 1	X	2006	Niger	181 [181]	P1.5–1,10–1	v.1 ID 74	75
MenX 2	X	2010	Burkina Faso	181 [181]	P1.5–1,10–1	v.1 ID 74	69
MenC	C	2007	Germany	NA^e^ [32]	P1.7,16	v.1 ID 1	ND^c^

^a^CC: Clonal Complex; ST: Sequence Type.

^b^Determined by sequencing of the fHbp gene and analysing the protein sequence using the Neisseria.org database [25].

^c^fHbp expression was measured by Western blot of whole cell lysates and in relation to group B strain H44/76, as previously described [21].

^d^ND: Not Determined.

^e^NA: Not Assigned.

### Statistical analysis

For statistical analysis, SBA titres <10 were assigned the value 1. Pearson’s Correlation test was used to evaluate linear correlation between two sets of values. Mann–Whitney U test was used to compare pairs of values. P ≤0.05 was considered statistically significant. Analysis was performed with GraphPad Prism 6 software.

## Results

### Pairwise comparison of fHbp-fH contact residues in 383 fHbp v.1 sequence IDs

At the time of the study, 383 fHbp v.1 peptide sequences were available in the public database (Neisseria.org) [25]. The 48 amino acids identified as contact sites with fH in each of the 383 IDs (S1 Table) were compared by sequence alignment. We created a matrix with the number of amino acid differences in the fH contact site between each pair of fHbp IDs. A section of the pairwise comparison matrix is shown in Fig 1 (full matrix in S2 Table). We identified 74 individual sequences of fHbp-fH contact residues; 41 of those (55%) were unique to one fHbp ID. The most shared fH-binding site sequence was found in 83 out of the 383 peptides (21.7%) and hence represents a fH-binding site consensus sequence, which we hypothesized might contribute to the induction of anti-fHbp antibodies with broad ID cross-reactivity. If one difference at any position in the fH-binding site consensus sequence was allowed, the number of IDs sharing it increased to 122 (31.9%). The biggest difference in the fH-binding site between any pair of IDs was 13 amino acids (27.1%), and was found between 8 different pairs of IDs. The biggest difference compared to the consensus sequence was identified among 5 fHbp IDs: ID 217 and ID 359 both had 10 amino acid (aa) differences compared to the consensus sequence (their fH-binding site being identical, and their whole peptide sequences differing by only one amino acid); ID 120 and ID 215 had 8 aa differences compared to the consensus sequence (their fH-binding site being identical, and their whole peptide sequences differing only in the N-terminal serine/glycine part); ID 55 similarly had 8 aa differences, compared to the consensus sequence, with its fH-binding sequence being one of the 41 unique IDs.

### Selection of fHbp IDs for over-expression on NOMV

To investigate whether there is a correlation between the sequence commonality of the fHbp region involved in binding fH, and the breadth of cross-protection induced by NOMV OE fHbp, against meningococcal isolates expressing different fHbp IDs, we selected different fHbp v.1 ID types for over-expression in *N*. *meningitidis* NOMV. We selected the fHbp peptides belonging to variant group 1, carrying either the fH-contact consensus sequence, or a less common sequence, and identified as the most prevalent among African case-related clinical isolates: ID 5, ID 9, and ID 74 [16, 30]. ID 9 is one of the peptides that contain the fH-binding site consensus sequence. We further included ID 1 among the peptides not carrying the consensus contact sequence, for comparison with previous work [21]. The fHbp-fH binding residues of the four selected fHbp ID types were all different from each other (Fig 1). Phylogenetic trees showing these four IDs in the context of the other 379 full length sequences, as well as of the other 70 fH-binding sequences, are represented in S1 Fig. fH-contact sequence of ID 1 is shared by a total of 17 fHbp v.1 peptides (4.4%), the sequence of ID 5 by 33 (8.6%), and sequence of ID 74 by 7 (1.8%) peptides. Among these four selected ID types, the minimum number of differences in the fHbp-fH contact sequence was 2 amino acids (4.2%) between ID 1 and ID 5, and between ID 5 and the consensus sequence in ID 9, while the maximum number of differences was 6 (12.5%), between ID 74 and ID 5, and between ID 74 and the consensus sequence in ID 9. The number of amino acid differences compared with the fH-binding site consensus sequence for ID 1 was 4 (Fig 1).

### Characterisation of NOMV with over-expressed fHbp

**Fig 2:**
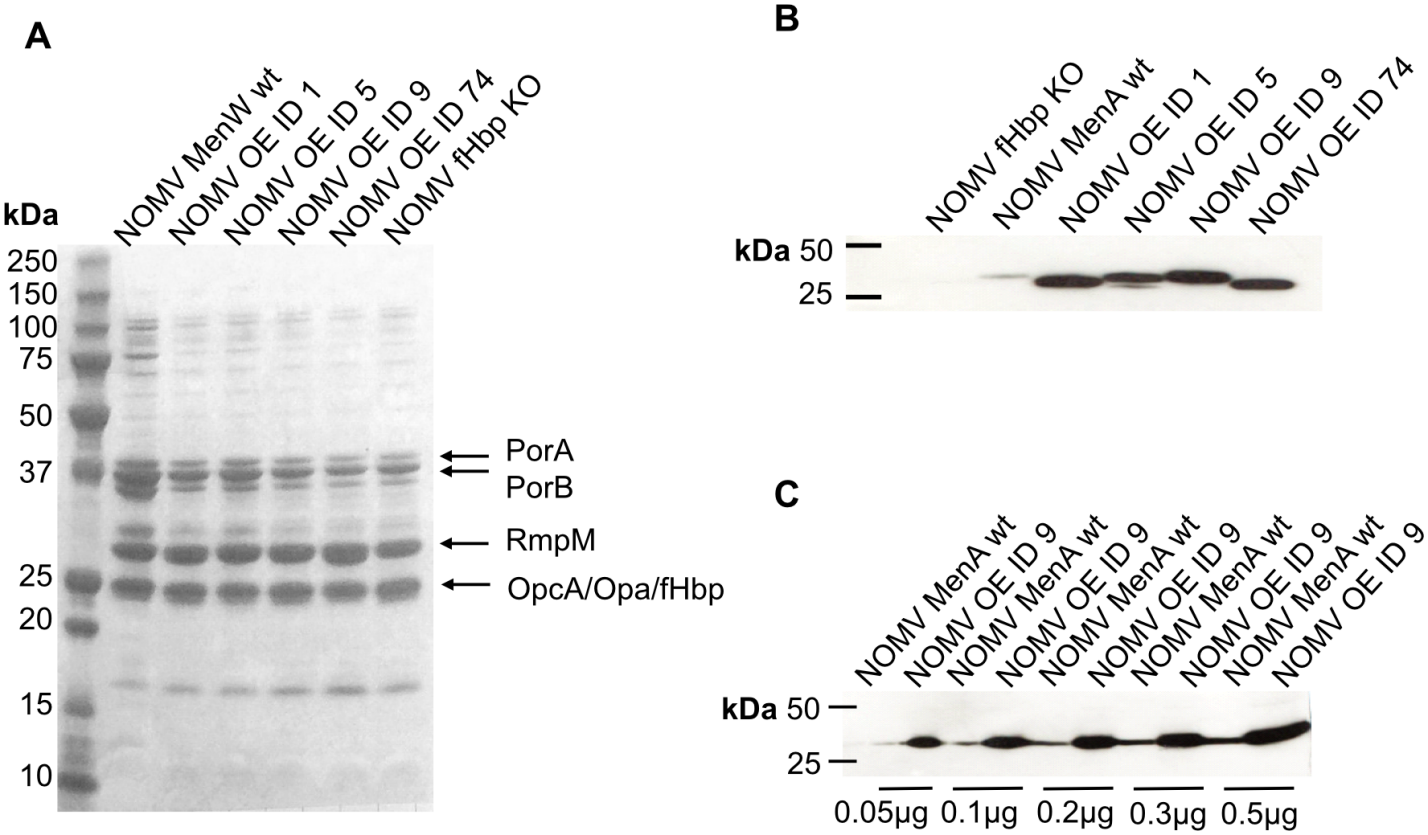
NOMV characterisation. (A) SDS-PAGE and Coomassie staining of NOMV. Samples: NOMV from MenW wild type; NOMV OE ID 1; NOMV OE ID 5; NOMV OE ID 9; NOMV OE ID 74; NOMV fHbp KO. 5 μg of each NOMV were loaded. The location of different proteins is indicated by the arrows. (B) Western blot to detect fHbp v.1 in NOMV from different mutants. As controls, NOMV fHbp KO and NOMV from MenA wild type (expressing fHbp ID 5) were also loaded. 0.2 μg of NOMV were loaded per lane. (C) Western blot for comparison of fHbp presence in NOMV from MenA wild type (expressing fHbp ID 5) and in NOMV OE ID 9. 0.05 to 0.5 μg of NOMV were loaded.

NOMV from the strains over-expressing the different fHbp IDs were prepared as described in the methods section. SDS–PAGE and Coomassie Blue staining revealed similar protein patterns in NOMV preparations from the different engineered meningococcal strains, consistent with the protein pattern of NOMV from the parental wild type strain (Fig 2A). NOMV from the mutant strains showed similar average radius of 53–57 nm, and similar size distribution, as determined by HPLC-SEC/MALS and dls (Table 4), in accordance also with micrographs obtained by Transmission Electron Microscopy.

**Table 4:** NOMV size characterisation by HPLC-SEC/MALS and dls.

	MALS	dls
	Radius, nm	Z-av (diameter, nm) (PdI)
NOMV OE ID 1	57.3	108.7 (0.331)
NOMV OE ID 5	53.6	91.8 (0.208)
NOMV OE ID 9	52.8	83.4 (0.145)
NOMV OE ID 74	56.0	106.9 (0.297)

Detection of fHbp in NOMV by Western blot, using a mouse polyclonal anti-fHbp v.1 antibody, indicated a comparable amount of fHbp present in NOMV from the different mutants (Fig 2B). This suggests that similar quantities of the four fHbp IDs were used for immunisation with the different NOMV, which is around 8.5% total protein content, as measured by Selected Reaction Monitoring (SRM, [7]). Comparison of fHbp levels in NOMV from MenW OE ID 9 and from a wild type MenA strain expressing medium levels of fHbp ID 5, showed that the level of fHbp detected in 0.05 μg of NOMV from the mutant is higher than that detected in 0.5 μg of NOMV from the wild type MenA (Fig 2C). This suggests that the mutant strains produce NOMV with more than 10-fold higher amounts of fHbp than this wild type strain.

### Anti-fHbp antibody response as analysed by ELISA

**Fig 3:**
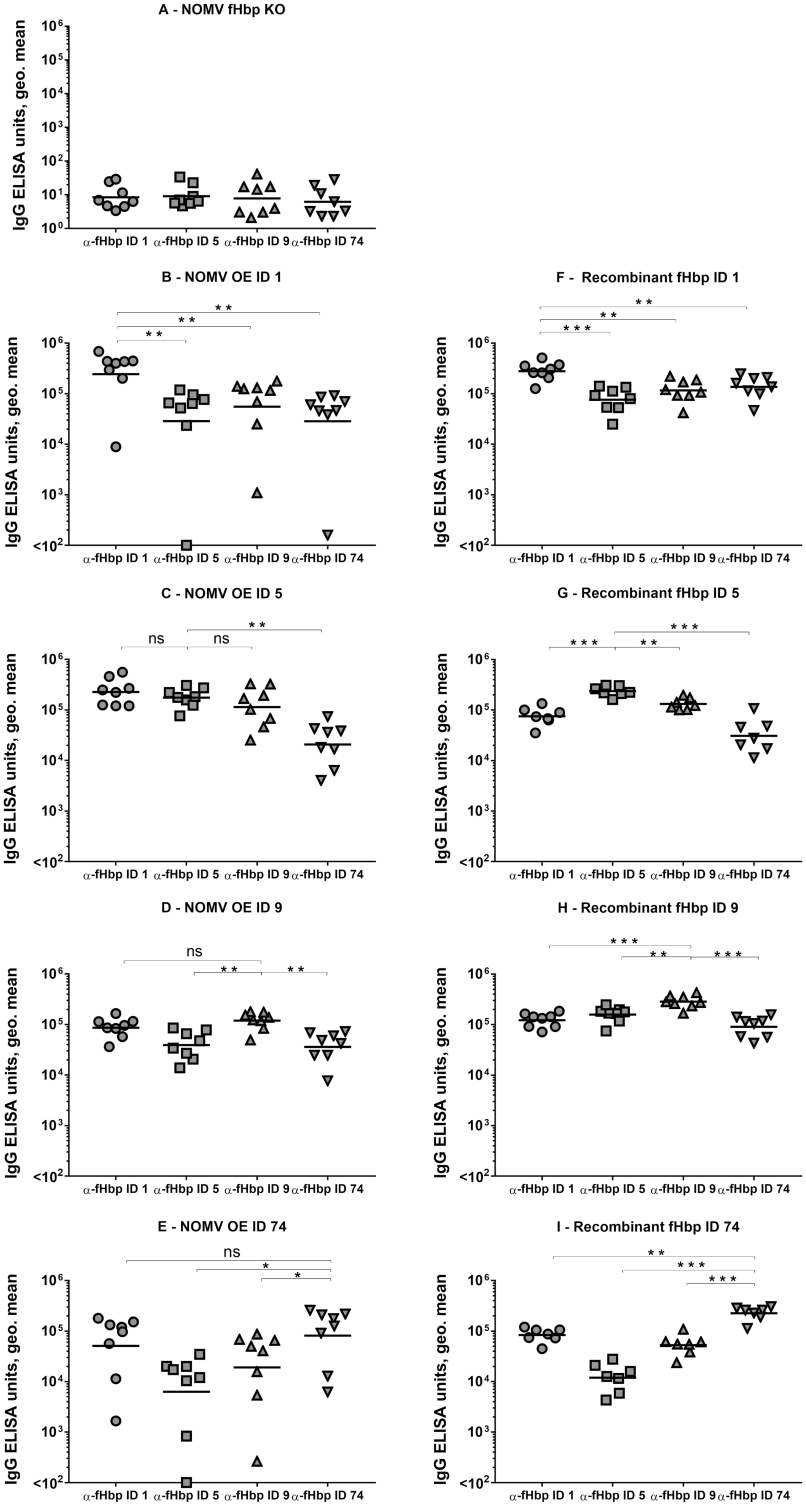
Anti-fHbp IgG antibody levels in sera from mice immunised with NOMV or recombinant fHbp. Anti-fHbp IgG antibody levels were measured in all immunisation groups against each of the 4 fHbp IDs. 1 μg/mL of recombinant fHbp was used for ELISA plate coating. Mice were immunised twice, four weeks apart, with 5 μg NOMV fHbp KO (A), or 1 μg NOMV OE one of the four different fHbp IDs (B-E), or with 20 μg recombinant fHbp (F-I). Serum samples analysed were obtained 2 weeks after the second dose. Each symbol represents a serum sample from an individual mouse; bars represent the geometric mean of each group. Mann-Whitney test 2-tailed was performed to compare pairs of groups: * *p* < 0.05, ** *p* < 0.01, *** *p* < 0.001.

To investigate the specificity and cross-reactivity of the anti-fHbp antibody response induced by NOMV OE the four diverse fHbp sequences, we compared IgG antibody levels against different recombinant fHbp ID types, in single mouse sera from different immunisation groups. Mice were immunised with either 1 μg of NOMV over-expressing one of the four fHbp ID types selected (ID 1, ID 5, ID 9, or ID 74), or 5 μg of NOMV without fHbp (NOMV fHbp KO), or 20 μg of one of the four recombinant fHbp IDs. By ELISA, we measured anti-fHbp IgG antibodies in single sera obtained 2 and 4 weeks after the first dose, and 2 weeks after the second dose (antibody levels at 2 weeks post second dose are shown in Fig 3). The antibodies present in each serum sample were measured against each of the four fHbp ID types used for immunisation.

Sera from mice immunised with NOMV without fHbp had no detectable anti-fHbp response (Fig 3A). NOMV OE ID 1 induced anti-fHbp IgG antibodies that were significantly higher when measured against the homologous ID, than against the three heterologous fHbp peptides (Fig 3B). NOMV OE ID 9 and NOMV OE ID 74 elicited high IgG antibody levels that were not significantly different when measured against the respective homologous fHbp IDs and fHbp ID 1, (Fig 3D and 3E). NOMV OE ID 5 induced IgG antibody levels against the homologous ID 5 and against heterologous ID 1 and ID 9 that were not significantly different. Altogether these data suggest that NOMV OE the different fHbp sequences induce a range of anti-fHbp IgG which collectively are able to bind the homologous and heterologous fHbp peptides, with NOMV OE ID 5 having reduced specificity compared to the others.

Compared with mice immunised with NOMV OE fHbp, mice immunised with recombinant fHbp consistently had IgG levels significantly higher when measured against the respective homologous fHbp sequences, than against any of the heterologous peptides (Fig 3F–3I). This finding confirms that anti-fHbp IgG elicited by fHbp on NOMV, compared to recombinant fHbp, are less specific for individual fHbp ID type and more broadly cross-reactive.

**Fig 4:**
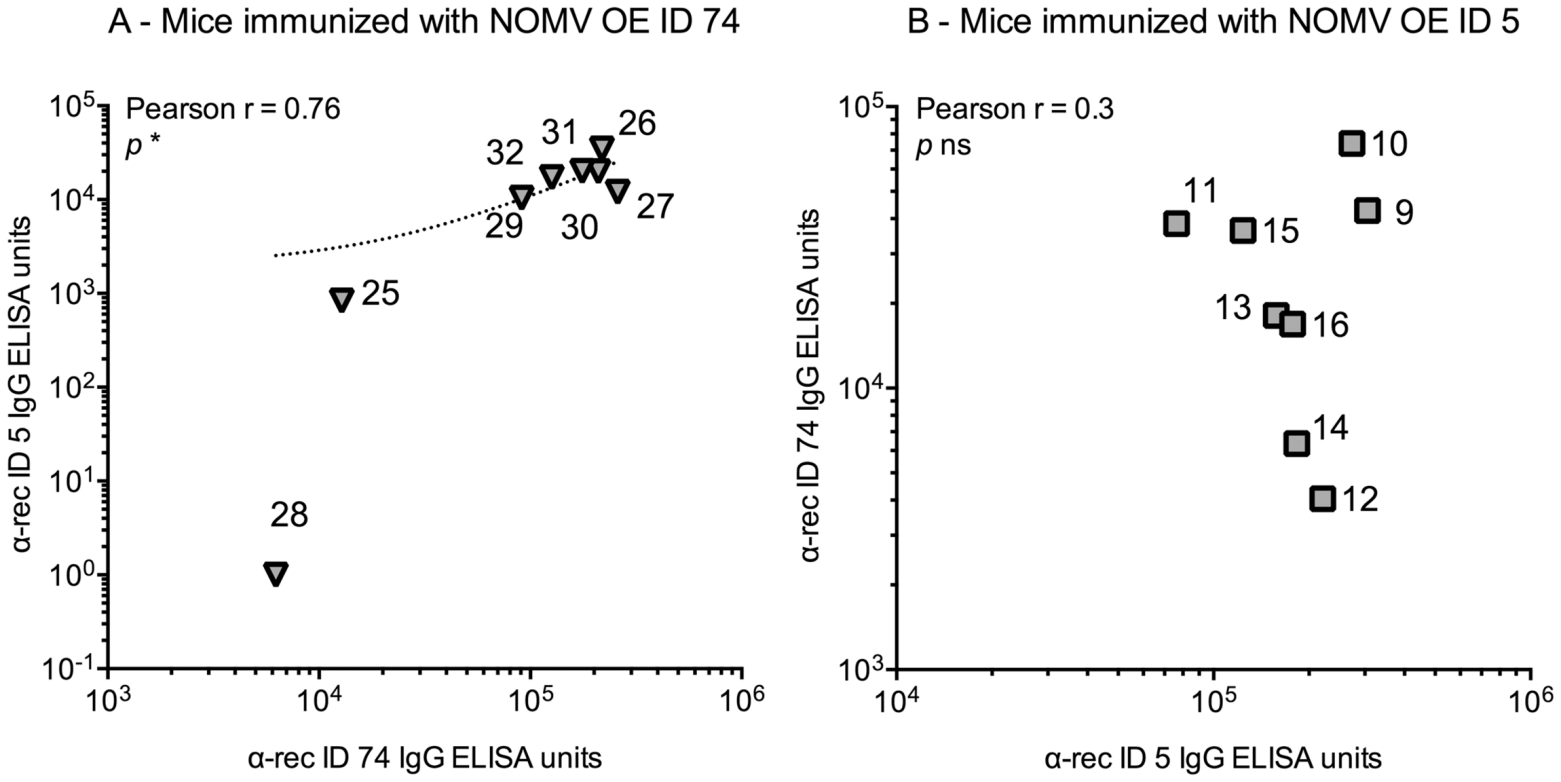
Comparison of IgG antibody levels against different fHbp ID within the same immunisation group. Comparison of IgG antibody levels against different recombinant fHbp IDs, in individual mice. Each symbol represents an individual mouse, identified by a number; 1 μg/mL of recombinant fHbp was used for ELISA plate coating. A) Mice immunised with NOMV OE ID 74 show linear correlation between anti-fHbp ID 74 IgG ELISA units (X axis) and anti-fHbp ID 5 IgG ELISA units (Y axis), by the Pearson Correlation test. B) Mice immunised with NOMV OE ID 5 do not show linear correlation between anti-fHbp ID 5 IgG ELISA units (X axis) and anti-fHbp ID 74 IgG ELISA units (Y axis).

We investigated if there was a correlation between IgG levels against pairs of different fHbp IDs, within each immunisation group. We found a correlation for 18 out of 24 comparisons for the groups immunised with NOMV OE fHbp, and 20 out of 24 comparisons for the groups immunised with recombinant fHbp. Correlation was not always reciprocal: in mice immunised with NOMV OE ID 74, for example, IgG antibodies against fHbp ID 74 and against fHbp ID 5 showed linear positive correlation (Pearson *r* = 0.76; *p* < 0.05), while this was not true for sera from mice immunised with NOMV OE ID 5, when tested against the same antigens (fHbp ID 5 and ID 74) (Fig 4; whole analysis in S2 Fig). Interestingly, sera from mice immunised with NOMV OE ID 9 showed a correlation for only two out of six possible combinations. The data indicate that different fHbp sequences induce antibodies with varying fine specificities in relation to binding to different fHbp IDs.

### SBA responses of mice immunised with NOMV with over-expressed fHbp variant group 1 IDs or recombinant fHbp

**Fig 5:**
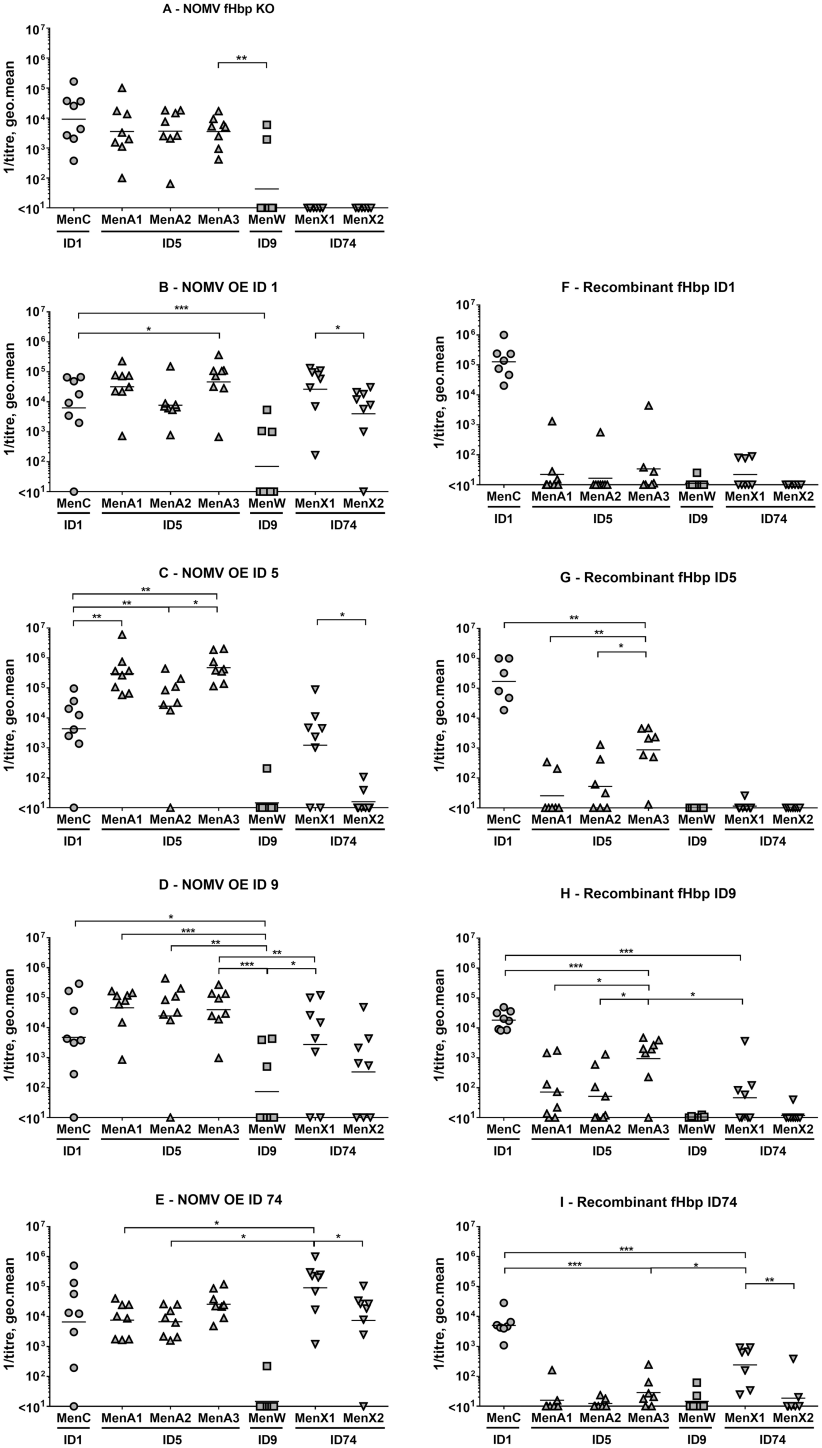
Serum bactericidal responses induced in individual mice immunised with NOMV or recombinant fHbp. Mice were immunised twice with NOMV (A-E), or with recombinant fHbp (F-I), four weeks apart, as described in the materials and methods. Serum samples obtained two weeks after the second dose were tested against a set of seven strains, expressing the four different fHbp ID types: ID 1 (●), ID 5 (▲), ID 9 (■), or ID 74 (▼). Each symbol represents the reciprocal titre of an individual mouse; horizontal bars represent geometric mean titres of the group.

We measured serum bactericidal antibody responses against diverse invasive meningococcal strains (Table 3), carrying each of the four fHbp IDs over-expressed in NOMV or purified as recombinant proteins, and carrying a heterologous PorA with respect to that present in the NOMV vaccines. We measured SBA titres in single sera from individual mice in each immunisation group (Fig 5, S3 Fig).

The MenC (ID 1) and MenA (ID 5) strains were susceptible to killing by sera raised against NOMV fHbp KO. Nevertheless, against strains carrying fHbp ID 5 (MenA1, MenA2, and MenA3) the highest SBA titres were induced by NOMV OE the homologous fHbp, even though for MenA1 and MenA2 these were not statistically different from SBA induced by NOMV OE ID 9.

Strains carrying fHbp ID 74 (MenX1 and MenX2) were resistant to sera raised against NOMV fHbp KO, and susceptible to killing by sera from mice immunised with NOMV OE each of the fHbp sequences (with the exception of MenX2, resistant to 6/8 sera raised against NOMV OE ID 5), indicating that killing of these strains is only due to anti-fHbp antibodies. Against both strains, the highest bactericidal responses were induced by NOMV OE homologous fHbp and fHbp ID 1, with titres significantly higher (*p* < 0.05) than those induced by NOMV OE fHbp ID 5.

The MenW strain used in the SBAs, is a low expressor of fHbp ID 9, and was resistant to killing by most of the mice sera tested. In contrast, the MenC strain carrying fHbp ID 1 was susceptible to killing by 7/8 or 8/8 individual sera from each group.

In general, mice immunised with recombinant fHbp developed lower SBA responses, compared to mice immunised with NOMV OE fHbp, with a higher number of mice showing no detectable bactericidal activity. This was true for all the meningococcal strains tested, except for MenC, which, as for the NOMV vaccines, was susceptible to killing by all sera.

**Fig 6:**
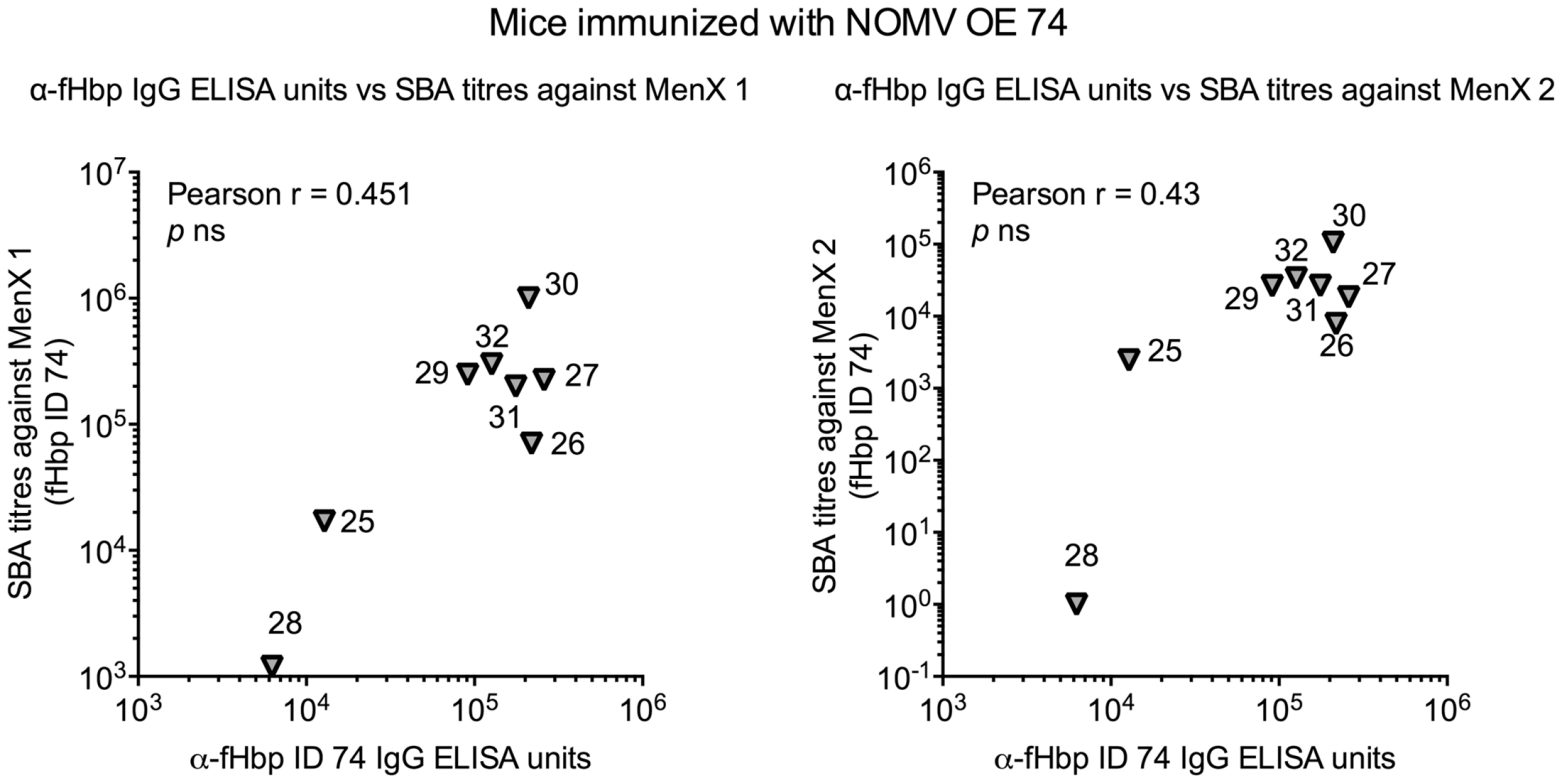
Correlation of IgG antibody levels against recombinant ID 74 and SBA titres against MenX strains. Each symbol represents a single mouse immunised with recombinant fHbp ID 74 and identified by a number. No linear correlation was evident according to the Pearson Correlation test.

Since MenX strains were resistant to sera raised against NOMV fHbp KO, we investigated whether a correlation exists between the SBA responses against these two strains and the anti-fHbp ID 74 IgG ELISA units, in mice immunised with NOMV OE fHbp ID 74. We found no correlation, suggesting that anti-fHbp IgG antibodies do not necessarily reflect the bactericidal activity of the sera analysed (Fig 6).

**Fig 7:**
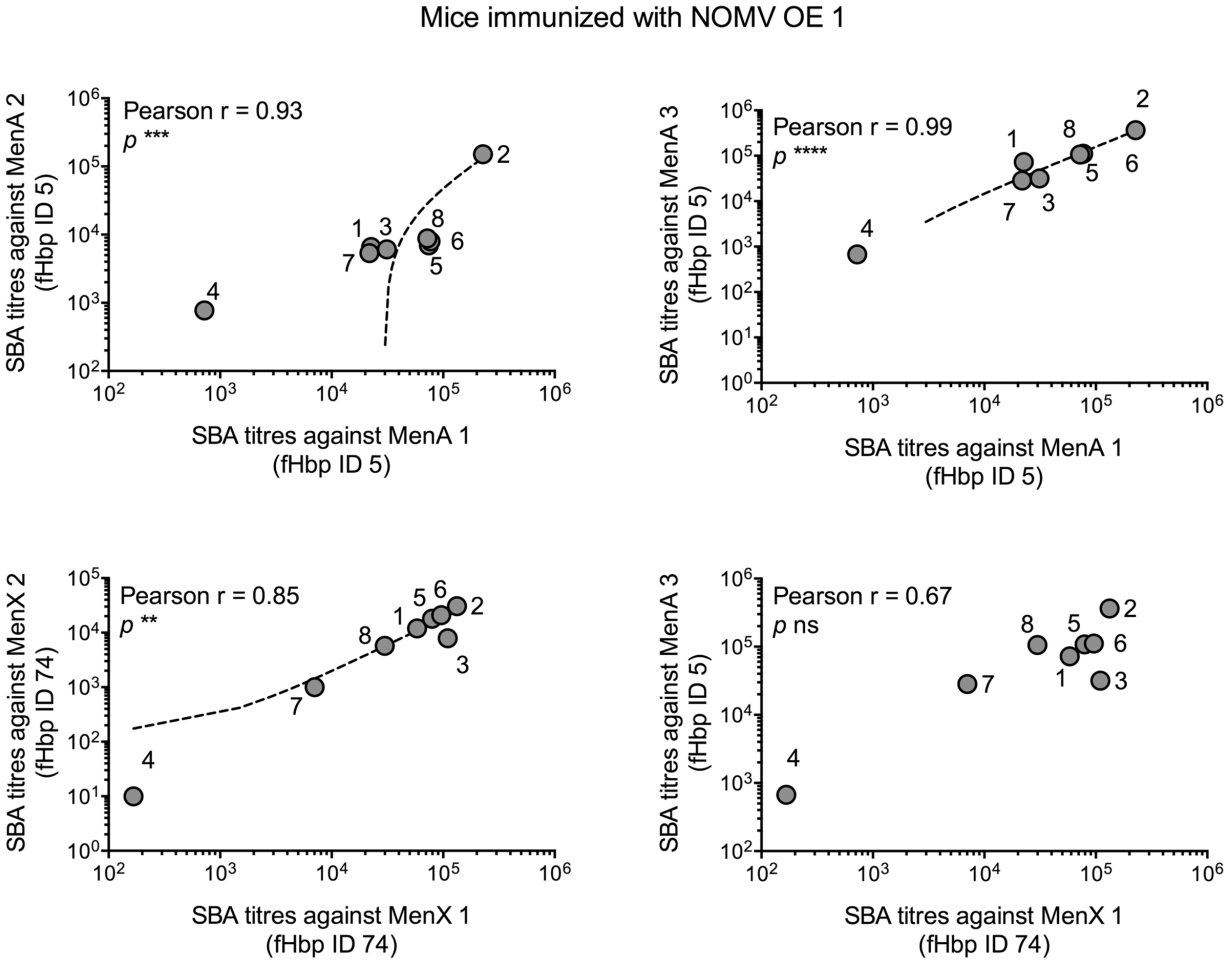
Correlation of SBA titres against different strains, in mice immunised with NOMV OE ID 1. Each symbol represents the reciprocal SBA titre of a mouse, identified by a number. According to the Pearson Correlation test, linear correlation is present between SBA titres against strains carrying the same fHbp ID, but not between SBA titres against strains carrying different fHbp ID.

Within each NOMV OE fHbp immunisation group, we compared bactericidal activity of individual sera against the various meningococcal isolates. In many cases, there was an absence of linear correlation between SBA titres against strains expressing different fHbp IDs, but we did find correlation between SBA titres against strains with the same fHbp ID. For example, in mice immunised with NOMV OE fHbp ID 1, SBA titres against two strains expressing either ID 5 or ID 74 correlated, but SBA titres against two strains expressing different fHbp IDs (e.g. one strain carrying ID 5 and one strain carrying ID 74) did not (Fig 7). The same was observed with NOMV with OE fHbp ID 5 and 74. This suggests that at least a subset of the functional antibodies elicited by NOMV OE fHbp target epitopes on fHbp that are ID-specific.

## Discussion

Factor H binding protein (fHbp) is a meningococcal virulence factor that can be successfully exploited as vaccine antigen [31, 32]. In mice, recombinant fHbp proteins generated antibodies with functional fine specificities against strains carrying fHbp IDs with a different sequence than the one used for immunisation; native outer membrane vesicles (NOMV) with over-expressed fHbp, instead, induced antibodies with higher cross-reactivity [3, 5, 6, 10, 16, 17, 21, 22].

Immunogenicity of NOMV with over-expressed fHbp ID 1 and ID 9 has been tested in mice in separate studies [21, 22, 33, 34]. However, no extensive analysis has been conducted so far to elucidate the specific contribution that the fHbp sequence IDs over-expressed on NOMV have on the generation of cross-reactive antibodies. We investigated the specificity and cross-reactivity of anti-fHbp antibody response elicited by NOMV with over-expressed different fHbp peptides belonging to variant group 1. Understanding the impact of fHbp sequence on the specificity and cross-reactivity of the immune response generated by NOMV OE fHbp, could guide the rational selection of a particular fHbp sub-variant to be expressed on NOMV, in order to broaden the potential coverage of a candidate vaccine. In the past, this relationship was investigated for recombinant fHbp: cross-reactivity induced by recombinant fHbp against strains expressing fHbp ID belonging to the same variant group is influenced by the ID sequence [3]. In particular, bactericidal activity induced by fHbp decreases with increasing genetic distance between fHbp sub-variant in the vaccine and on the strain tested [35], and IDs more central located by phylogenetic analysis can elicit broader cross-protective bactericidal responses, compared to IDs that are more peripheral [36]. However, analysing the entire fHbp sequence, and its variability among the different IDs, may not generate adequate information for our investigation: with such analysis, variations at positions not necessarily immunologically relevant are taken into consideration. Therefore, without discounting that other parts of fHbp, and their variations, can be important as targets of bactericidal antibodies, we chose to focus our attention on the surface-exposed amino acids of fHbp involved in factor H (fH) binding. We hypothesized that variations in this region of the molecule are more critical for the generation of cross-reactive antibodies, compared to variations of amino acids in other part of the protein, and not surface-exposed; such consideration was also suggested for recombinant fHbp [36]. As a consequence, it is possible that there is a fH-contact sequence that is conserved among different fHbp IDs, and that contributes to eliciting anti-fHbp antibodies with extensively broad ID cross-reactivity. Our analysis revealed a consensus sequence of the whole fH-binding site that is identical in 22% of the known fHbp ID sequences at the time of the analysis.

We tested the immunogenicity in mice of NOMV derived from an African W meningococcal strain, devoid of wild type v.2 fHbp and capsule biosynthesis, and over-expressing one out of four different fHbp v.1 ID types (ID 1, ID 5, ID 9, or ID 74), relevant among currently circulating African invasive isolates and/or expressed in NOMV in previous studies [16, 21, 30]. Among the fHbp IDs selected, ID 9 contained the fH-binding site consensus sequence.

NOMV OE fHbp induced similar anti-fHbp antibody levels compared to recombinant fHbp, even if the amount of fHbp administered with the NOMV vaccines was more than 230 times lower than that administered as recombinant protein. Nevertheless, each of the NOMV OE fHbp vaccines induced higher and broader serum bactericidal antibodies compared to the equivalent recombinant proteins; one element that may contribute to such an outcome is a synergistic effect of the antibodies against the wide range of antigens present on NOMV (e.g. PorA, PorB, Opa/Opc, FbpA, FetA, NadA [37–40]). In mice immunised with recombinant fHbp, Pajon et al. [16] observed high SBA responses against strains expressing the identical ID used for immunisation, but lower cross-reactivity against strain with different IDs, even if belonging to the same variant group. Against the MenA and MenX strains we observed similar trends, although we recorded lower SBA titres. It is remarkable that a considerable number of mice immunised with recombinant fHbp did not develop antibodies with serum bactericidal activity against most meningococcal strains tested. These mice are not non-responders, as evident from the ELISA data: they have developed anti-fHbp antibodies, but these antibodies are unable to kill meningococci efficiently. Intrinsic differences in the ability of the strains to survive in sera, especially combined with diverse fHbp expression levels, may contribute to differences reported here compared with other studies. For example, the MenW strain used in the SBAs expressed very low levels of fHbp, likely explaining its resistance to killing by sera containing antibodies targeted to fHbp.

Nevertheless, our data indicate that anti-fHbp IgG ELISA titres do not reflect the bactericidal activity of the sera, even when such activity appears to be dependent on anti-fHbp antibodies. This lack of linear correlation contrasts to what has been observed in adult humans for anti-DOMV IgG [41, 42] and anti-serogroup C capsule IgG [43]. Several factors may account for this difference: i) differences between human and murine immune system; ii) antibodies directed towards a wider range of antigens elicited by NOMV compared to DOMV; iii) the different nature of the antigens themselves, particularly in the case of MenC polysaccharide and fHbp (a polysaccharide and a protein, respectively). The fact that anti-fHbp IgG antibodies elicited by NOMV do not necessarily reflect the bactericidal activity of sera following immunisation with NOMV, perhaps highlights the breadth of protective antigens present on NOMV. In contrast, the lack of correlation between ELISA for fHbp and SBA when bacterial killing appears only to be dependent on anti-fHbp antibodies, is surprising. This suggests that fHbp can elicit an IgG antibody response against non-protective epitopes not only when administered as recombinant proteins, but also when presented on NOMV.

Nevertheless, compared to recombinant fHbp, fHbp on NOMV appears to be able to elicit antibodies directed against protective epitopes, not only present on the homologous ID peptide, but also on heterologous ID peptides. An important consideration in relation to this is the presentation of antigens on NOMV in a more native environment and native conformation, compared to recombinant proteins, so that the epitopes on the NOMV vaccines better resemble those of the bacterium. Moreover, the intrinsic adjuvanticity of outer membrane vesicles is also relevant [44, 45]: the immune response against protective antigens on NOMV is likely to be enhanced due to the stimulation of innate receptors (e.g. TLR2 by PorB, TLR4 by LOS [46, 47]), and may also be affected by a different balance in IgG subtype induced by NOMV OE fHbp, compared to the recombinant protein [48].

Most meningococcal vaccine studies analyse the serum bactericidal response against *N*. *meningitidis* testing pools of sera. However, with this kind of analysis, it is not possible to appreciate individual differences, which become hidden in the pools, resulting in potentially misleading immune response readouts. Therefore, we performed the SBA assays by testing single mouse sera. This approach, however, had the consequence of only being able to test a limited number of strains in SBAs (because of resource constraints), and with rabbit, not human, complement (because of the large amount of complement required). Nevertheless, we can now appreciate small differences in the anti-fHbp antibody response generated by similar but different vaccine antigens, as well as the individual variations in response within groups of animals. These results suggest that even when expressed on NOMV, where plenty of other antigens contribute to the bactericidal response elicited, the fHbp sequence has an impact on the cross-reactivity of the antibodies induced. We did not expect anti-PorA (P1.5,2) antibodies generated by the NOMV vaccines to contribute to SBA activity against the strains tested, as these were chosen to have heterologous PorA molecules. Data confirmed this, also in the case of different variant regions (VR) belonging to the same family, between the PorA of the tested strain and the one of the vaccine: the MenX strains expressing PorA VR1 5–1 were completely resistant to killing by antibodies against NOMV fHbp KO, and the group W strain expressing PorA VR2 2–36 was resistant to most of the sera raised against the NOMV vaccines (likely secondary to low amounts of fHbp expression by this strain). SBA results suggest that each fHbp ID over-expressed on NOMV elicits bactericidal antibodies directed towards ID-specific epitopes on the fHbp molecule that differ for different ID types. This is consistent with ELISA results, which indicate that some fHbp IDs induce antibodies against more conserved and shared epitopes of the fHbp molecule, while others induce antibodies against more variable epitopes.

Our data do not show an obvious correlation between similarities in the fH-contact sequence of the fHbp over-expressed on NOMV, and breadth of cross-reactive antibodies generated. However, the overall high antibody responses induced by NOMV OE fHbp and serum bactericidal activity elicited by NOMV fHbp KO represent a limitation: they mask small differences in magnitude and functionality of antibody responses to fHbp generated among immunisation groups. Moreover, as the IDs selected for over-expression differ not only in the fH-binding site, but also in other parts of the protein sequence, the dissection of the specific relevance of fH-contact residues is not straightforward. Therefore, it would be informative to engineer investigational NOMV vaccines, expressing fHbp IDs with different fH-binding sites, but identical with respect to the rest of the fHbp amino acid sequence. Another interesting approach would be to expand the analyses to residues of fHbp surrounding fH-contact site. In this regard, however, our analysis already has considered, among fH-contact residues, extra amino acids, with respect to previous studies [49].

In conclusion, our study confirms that, in contrast to recombinant fHbp, NOMV OE fHbp generate a broad bactericidal antibody response that is able to kill heterologous strains of meningococcus. Differences in the fHbp sequence expressed on NOMV can contribute to different fine specificities in the antibody response generated. We provide a new structure-sequence approach for the rational selection of fHbp IDs to be used in NOMV-based vaccines, focusing attention on functional surface-exposed regions. Focusing on common motives in surfaced-exposed crucial sites, could be valuable for elucidating the contribution of other protective antigens to the cross-reactivity against meningococcal strains, elicited by outer membrane vesicles. This would be extremely valuable for the design of future native outer membrane vaccines. Finally, the same technology could be applied to the development of other vaccines.

## Supporting information

S1 Fig Phylogenetic analyses among fHbp sequence variants. A) Phylogenetic tree built with the 383 fHbp v.1 IDs whose fH-binding site was analysed in S1 Table. The four IDs selected as investigational vaccines in the present study are indicated. B) Phylogenetic tree built with the 74 fH-binding site sequences, identified among the 383 v.1 fHbp IDs analysed. The sequences carried by the four IDs selected as investigational vaccines in the present study are indicated. Bars represent substitutions per site.
(TIF)

S2 Fig Comparison of IgG antibody levels against different fHbp ID, within a same immunisation group. Comparison of IgG antibody levels against different recombinant fHbp IDs, in individual mice. Each symbol represents an individual mouse, identified by a number.
(DOCX)

S3 Fig Serum bactericidal responses against each of the seven meningococcal strains, induced by the different immunisations. SBA titres presented in Fig 5 are here represented highlighting how the seven meningococcal strains tested are differently killed by all the sera analysed. Each graph represents a strain tested; immunisation groups are indicated on X axes. Each symbol represents the reciprocal titre of an individual mouse; horizontal bar represents geometric mean titres of the group. The Mann-Whitney test 2-tailed test was performed to compare pairs of groups; p < 0.05 was considered significant: *p < 0.05; **p < 0.01; ***p < 0.001.
(TIF)

S1 Table fH-contact residues in fHbp variant 1 peptides. (XLSX)

S2 Table Pairwise comparison of number of different residues in fH-contact sequence of variant 1 fHbp peptides. (XLSX)
