# Supplementary figures and images for: Contribution of factor H-Binding protein sequence to the cross-reactivity of meningococcal native outer membrane vesicle vaccines with over-expressed fHbp variant group 1
Source: PLoS One. 2017 Jul 25;12(7):e0181508. doi: 10.1371/journal.pone.0181508 (PMC5526518; doi:10.1371/journal.pone.0181508)

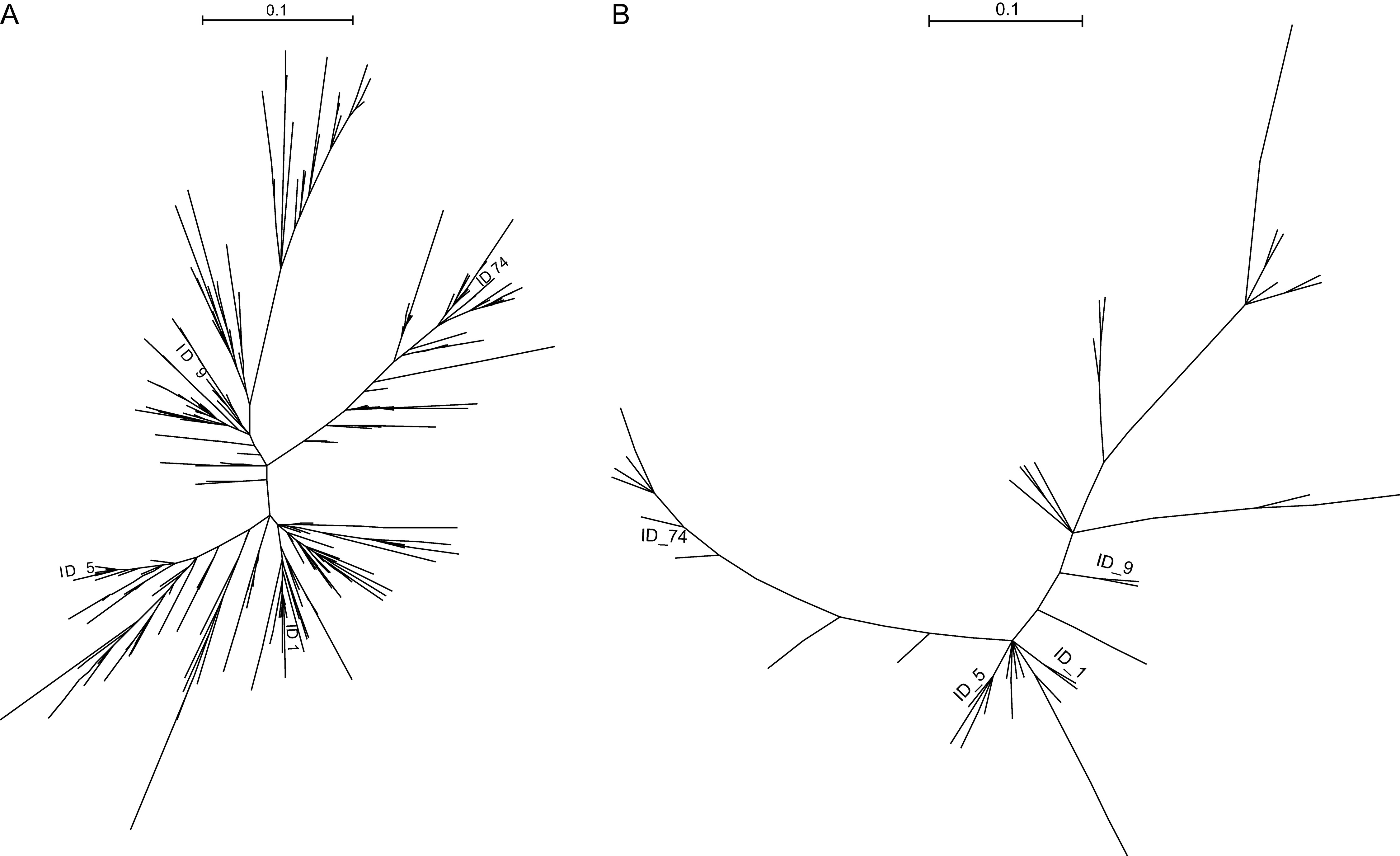

Supplement: S1 Fig — A) Phylogenetic tree built with the 383 fHbp v.1 IDs whose fH-binding site was analysed in S1 Table. The four IDs selected as investigational vaccines in the present study are indicated. B) Phylogenetic tree built with the 74 fH-binding site sequences, identified among the 383 v.1 fHbp IDs analysed. The sequences carried by the four IDs selected as investigational vaccines in the present study are indicated. Bars represent substitutions per site. (TIF) [file pone.0181508.s001.tif]

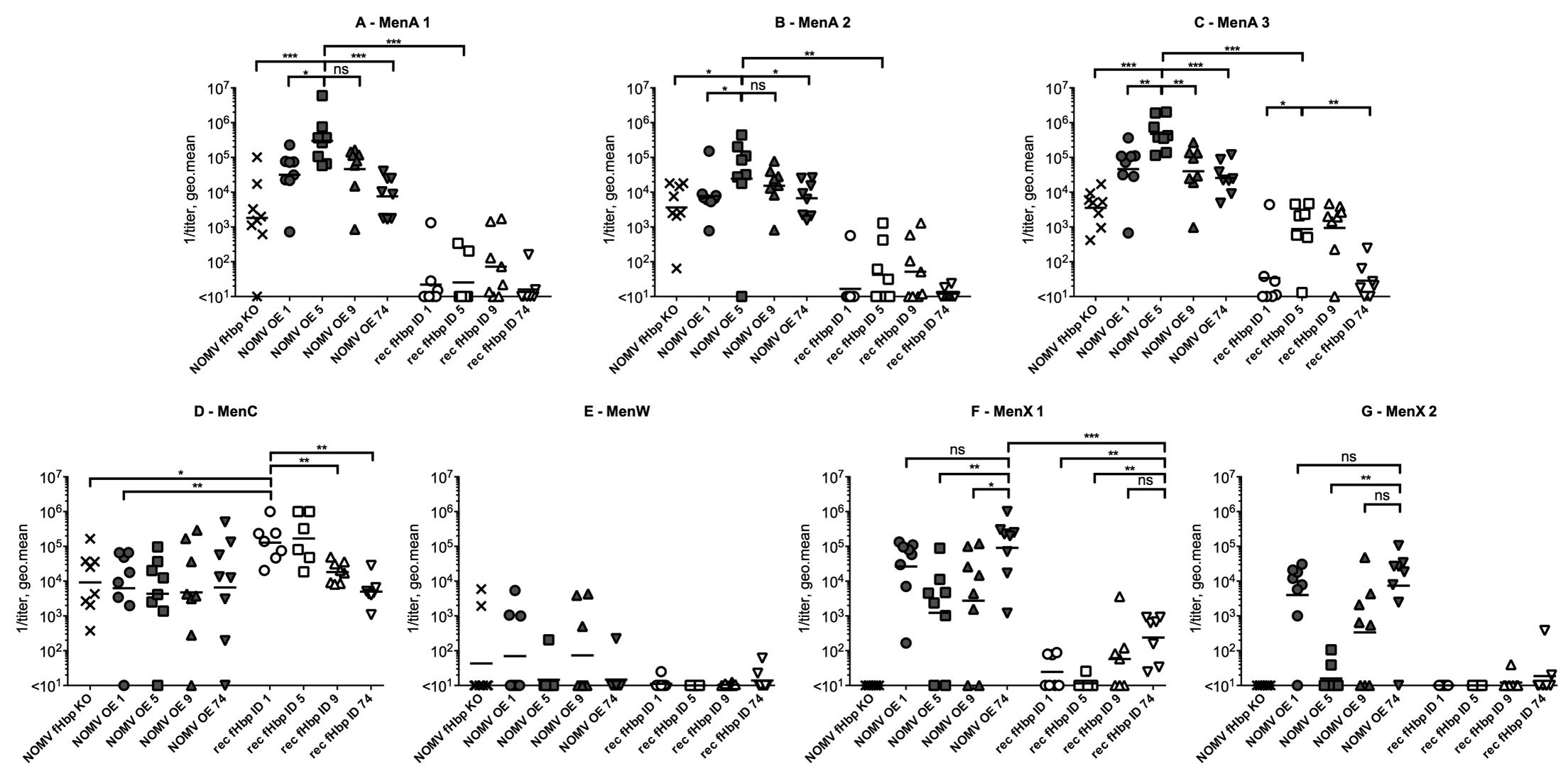

Supplement: S3 Fig — SBA titres presented in Fig 5 are here represented highlighting how the seven meningococcal strains tested are differently killed by all the sera analysed. Each graph represents a strain tested; immunisation groups are indicated on X axes. Each symbol represents the reciprocal titre of an individual mouse; horizontal bar represents geometric mean titres of the group. The Mann-Whitney test 2-tailed test was performed to compare pairs of groups; p < 0.05 was considered significant: *p < 0.05; **p < 0.01; ***p < 0.001. (TIF) [file pone.0181508.s003.tif]
