# Supplementary material for: Contribution of factor H-Binding protein sequence to the cross-reactivity of meningococcal native outer membrane vesicle vaccines with over-expressed fHbp variant group 1
Source: PLoS One. 2017 Jul 25;12(7):e0181508. doi: 10.1371/journal.pone.0181508 (PMC5526518; doi:10.1371/journal.pone.0181508)

S2 Figure. Comparison of IgG antibody levels against different fHbp ID, within a same immunization group.

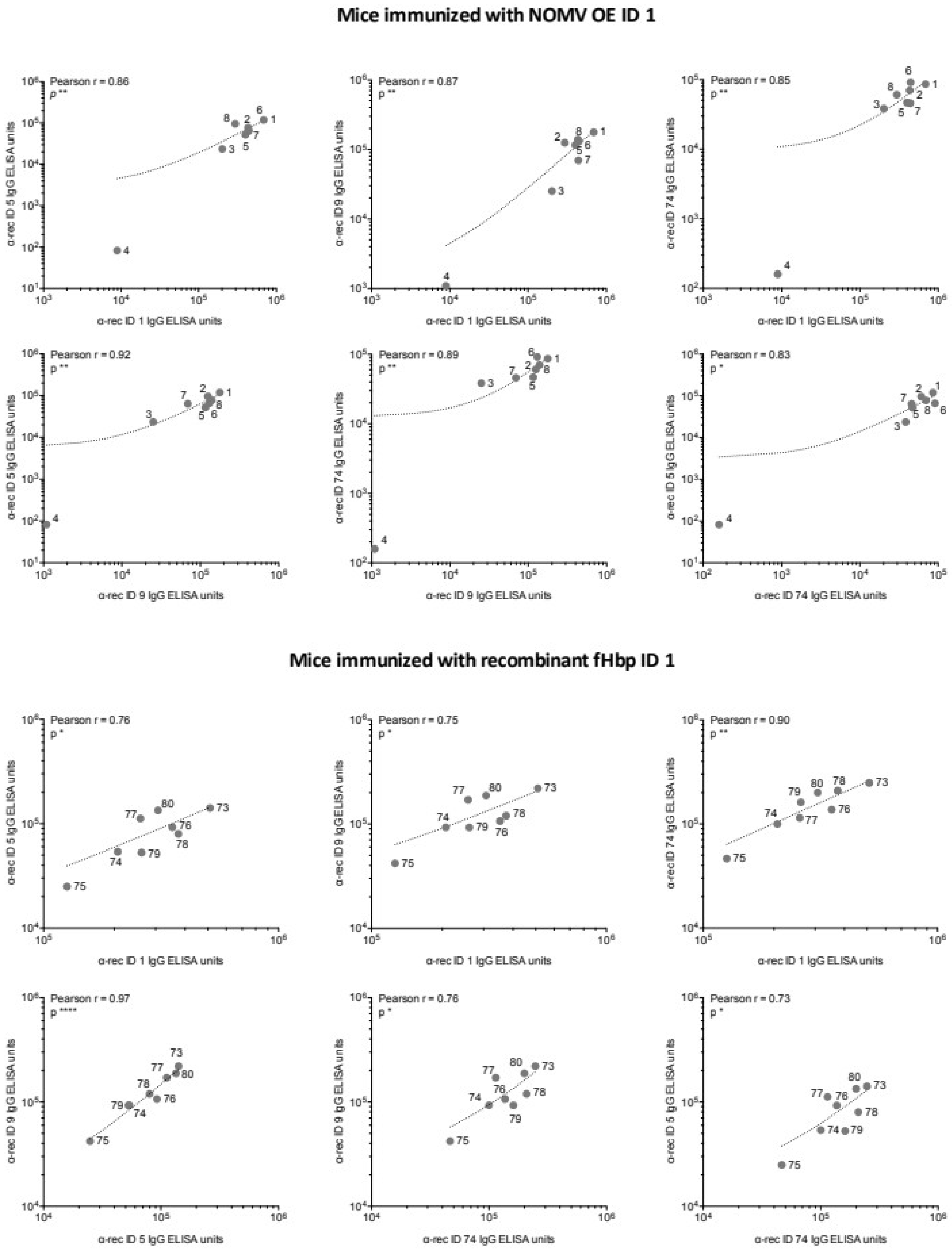

S2 Figure. Comparison of IgG antibody levels against different fHbp ID, within a same immunization group.

Mice immunized with NOMV OE ID 5

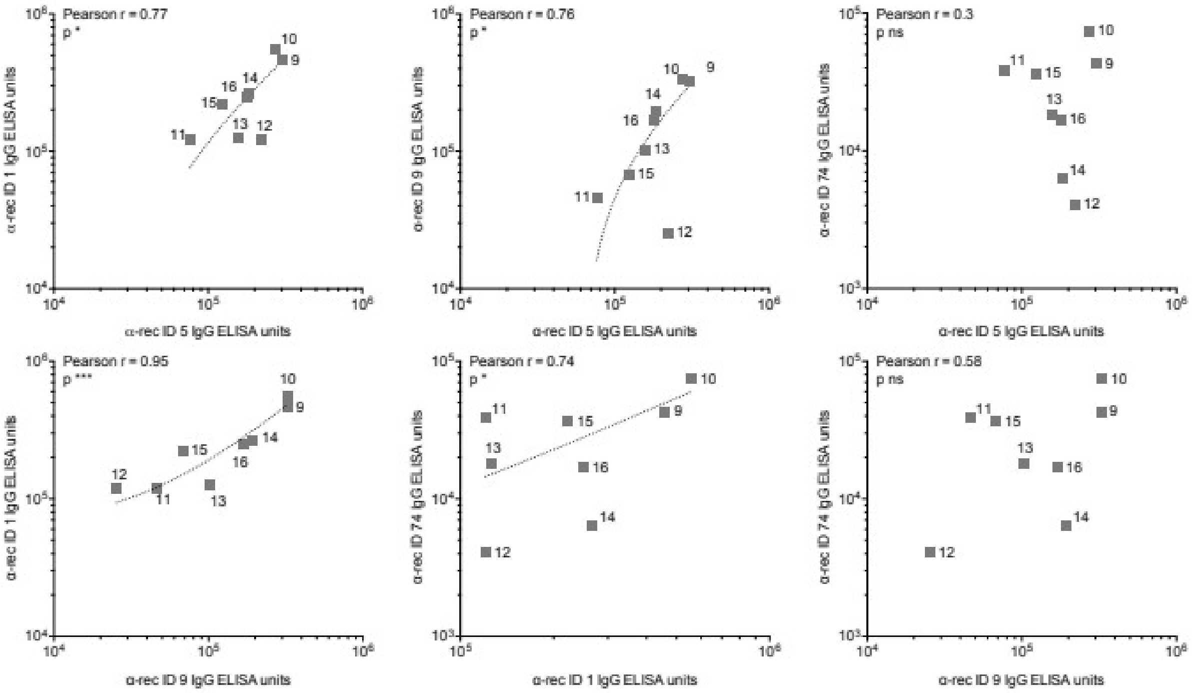

Mice immunized with recombinant fHbp ID 5

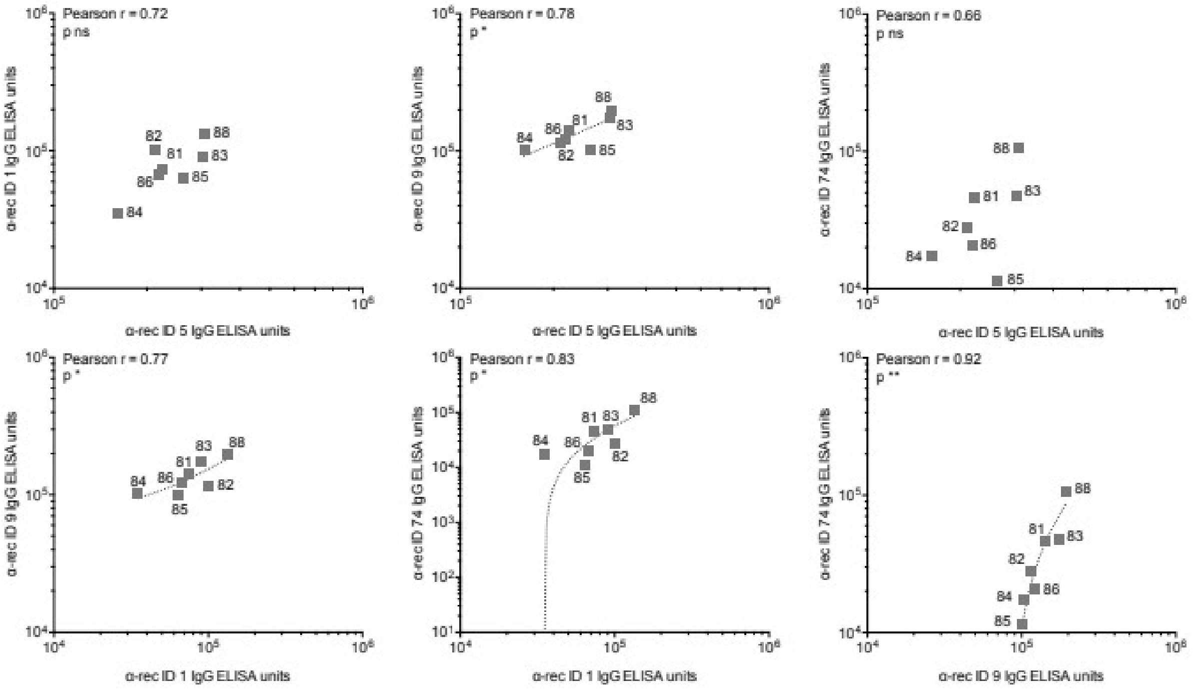

S2 Figure. Comparison of IgG antibody levels against different fHbp ID, within a same immunization group.

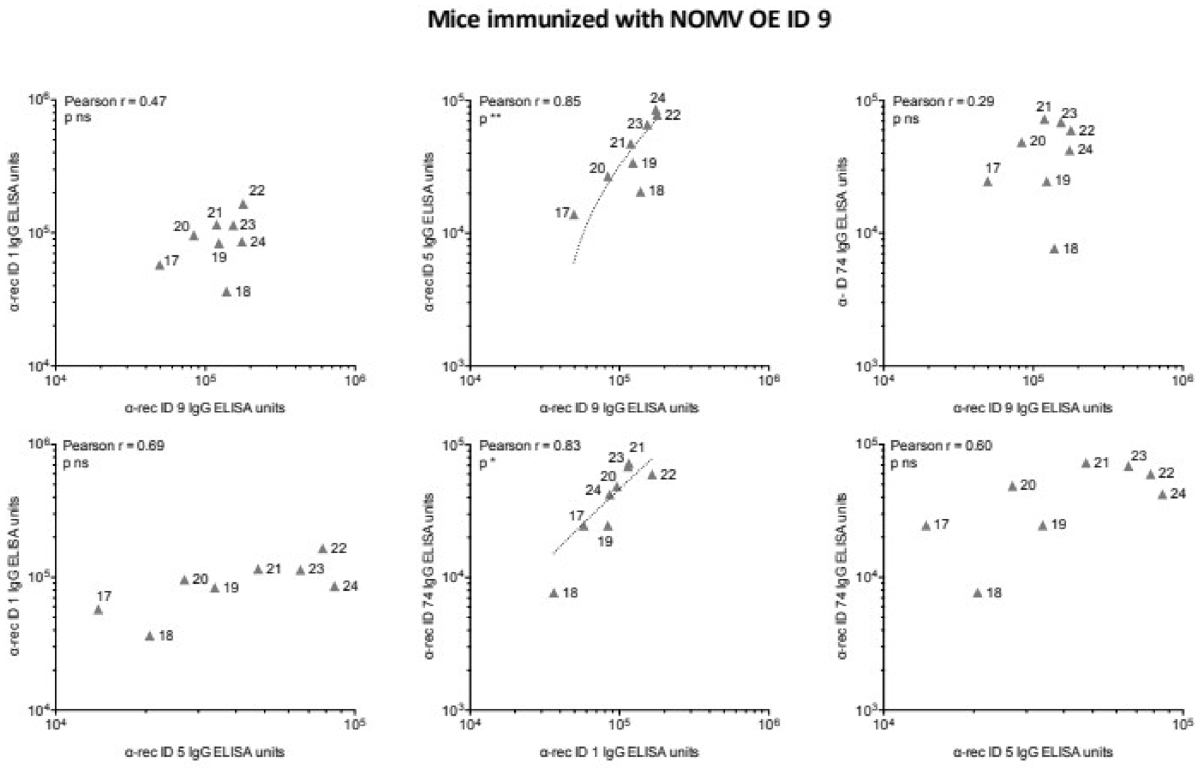

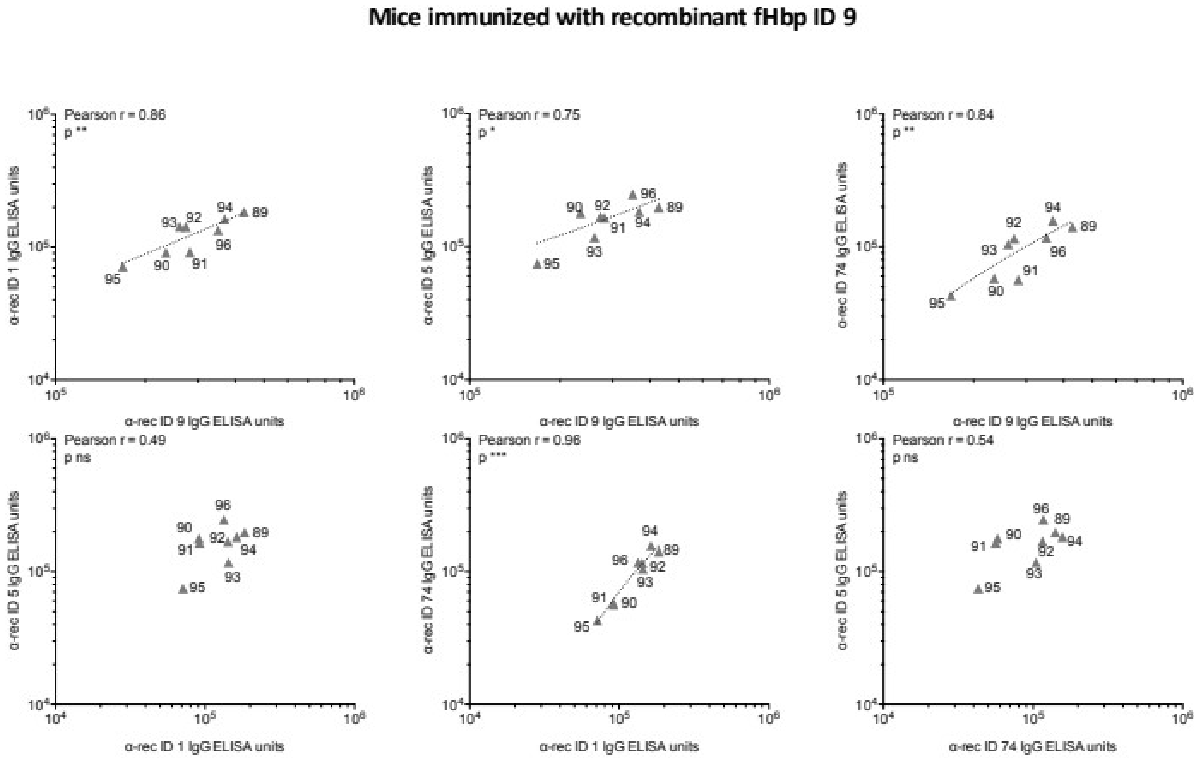

S2 Figure. Comparison of IgG antibody levels against different fHbp ID, within a same immunization group.

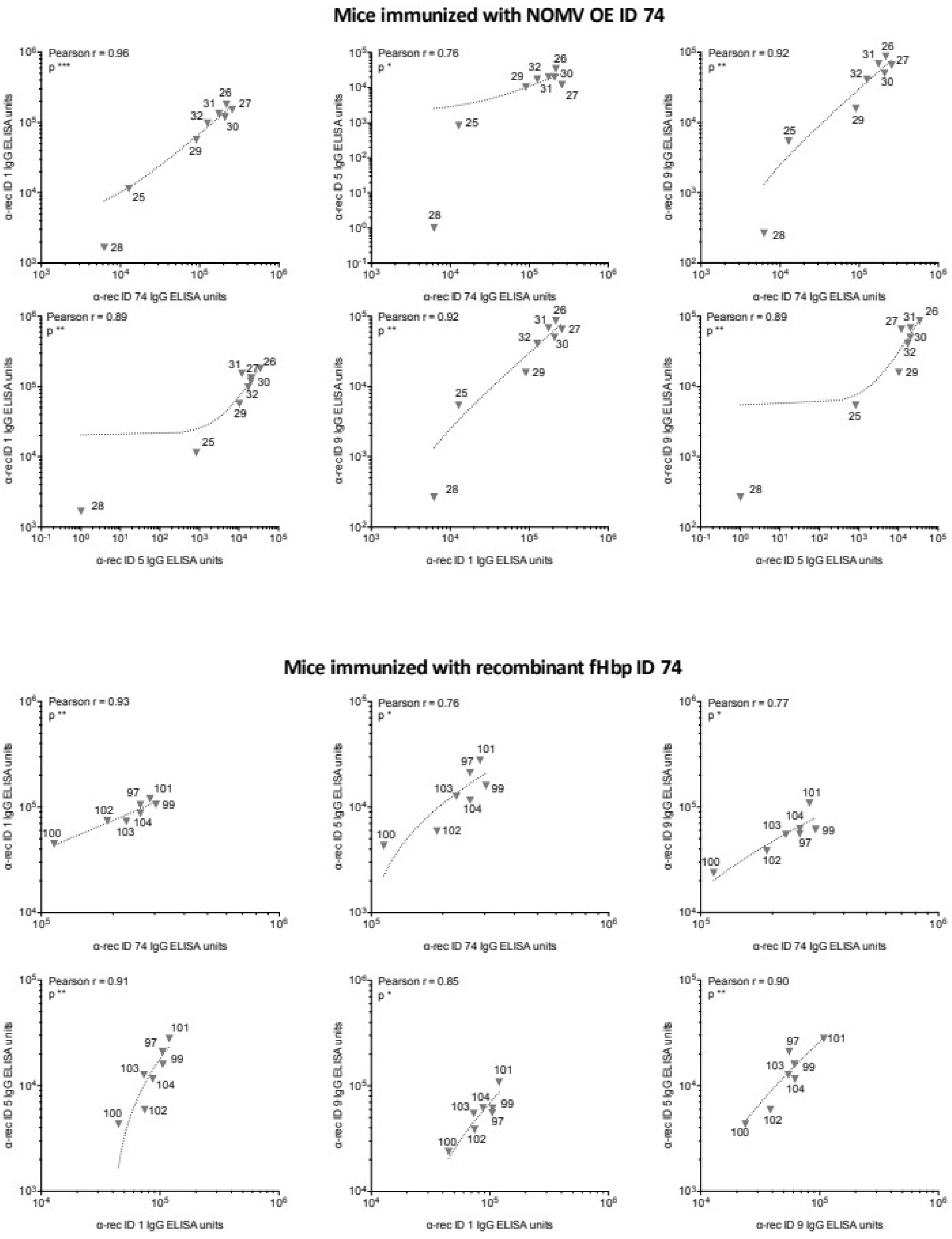

Supplement: Supplementary file 2 [file pone.0181508.s002.docx]
